# Effects of Velocity-Based French Contrast Training on Lower-Limb Power and Delivery Kinetics in Medium-Fast Cricket Bowlers: A Randomized Controlled Trial

**DOI:** 10.3390/sports14060226

**Published:** 2026-06-01

**Authors:** Qidong Zhao, Chunlei Li

**Affiliations:** 1 Winter Sports Collage, Beijing Sport University, 48 Xinxi Road, Beijing 100084, China; 2 School of Strength and Conditioning, Beijing Sport University, 48 Xinxi Road, Beijing 100084, China; lichunlei1111@163.com

**Keywords:** strength and conditioning for cricketers, bowling performance, lower limb explosive power, delivery stride kinetics

## Abstract

The bowling performance of cricket fast bowlers is highly dependent on lower limb power and stiffness. French Contrast Training (FCT) and Velocity-Based Training (VBT) are effective ways to improve rate of force development and peak power. The objective of this study was to investigate the effects of VBT-optimized FCT on the lower limb explosive power and bowling performance of cricket fast bowlers. Twenty adult male medium-fast bowlers volunteered for this study and were evenly divided into an experimental group (EG) and a control group (CG). The EG underwent an 8-week VBT-based FCT program, while the CG completed 8 weeks of traditional resistance training combined with traditional plyometric training. Before and after the intervention, subjects were tested on their Bulgarian split squat load–velocity profile, general lower limb power (countermovement jump height, squat jump height, Eccentric Utilization Ratio, and Reactive Strength Index), and bowling performance metrics (front foot contact time, peak force, impulse, front knee angle at ball release, and ball release speed). The results demonstrated that the EG showing significant advantage over the CG on movement velocity during the Bulgarian split squat at loads 20% 1RM, 40% 1RM, and 60% 1RM (*p* = 0.008, 0.011, 0.008, $\eta_{p}^{2}$ = 0.337, 0.313, 0.324). General lower limb power in the EG also improved significantly, with CMJ height, EUR, and RSI showing significant inter-group superiority compared to the CG (*p* < 0.001, = 0.019, 0.004, $\eta_{p}^{2}$ = 0.659, 0.281, 0.399). Regarding bowling performance, the EG demonstrated highly significant advantages in front foot contact impulse, front knee angle at ball release, and ball release speed (*p* < 0.001, $\eta_{p}^{2}$ = 0.572, 0.590, 0.704). In conclusion, the 8-week VBT-FCT program is more effective than the traditional resistance and plyometric training program of the same duration in enhancing lower limb power and bowling performance for medium-fast cricket bowlers.

## 1. Introduction

Originating in England, cricket boasts a history spanning nearly 500 years. Alongside the historical expansion of the British Empire, the sport has disseminated globally, establishing itself as one of the most popular sports in the contemporary era. On-field positions in cricket are generally categorized into five primary roles: fast bowlers, spin bowlers, batters, wicket keepers, and fielders. Among these, the fast bowler plays a pivotal role within the team. An elite fast bowler delivers the ball with high velocity and pinpoint accuracy, significantly impeding the opposing batter’s ability to execute effective strokes. A high-velocity delivery can induce misjudgments from the batter, leading to missed strikes and subsequent dismissals [1]. The fast-bowling action can be broadly delineated into four phases: the run-up, take-off, landing, and delivery. During the run-up phase, kinetic energy is accumulated through forward acceleration. The take-off and landing phases require effective lower limb braking to redirect this forward kinetic energy into trunk rotation. Subsequently, the rotating trunk drives the arm to release the ball. It is crucial to note that cricket rules dictate that the bowler’s arm must remain straight during delivery; consequently, the ball release speed is heavily reliant on lower limb power and explosive trunk rotation capabilities [2]. Therefore, the maximal ball release speed achievable by a fast bowler is not dictated solely by their technical proficiency, but also significantly depends on their lower limb explosive power and rapid force production capabilities [3].

Explosive power can be defined as the ability to generate high forces in a short timeframe [4]. This capacity is paramount for athletes across a multitude of sports, including cricket fast bowlers. Consequently, numerous studies have explored effective methodologies for enhancing explosive power [5,6]. French Contrast Training (FCT) has emerged as a crucial component in this domain; rather than a single exercise, it is a highly structured training methodology. Originally pioneered by French track and field coach Gilles Cometti, it was further systematized and popularized by strength and conditioning coaches Cal Dietz and Ben Peterson in their seminal book, Triphasic Training [7]. FCT primarily comprises four distinct training components: (1) heavy resistance exercise, (2) bodyweight plyometric exercise, (3) plyometric exercise facilitating maximal power output, and (4) accelerated or assisted plyometric exercise. Within this sequence, exercises 1 and 2, as well as 3 and 4, respectively constitute complex training pairs; conversely, exercises 1 and 3, along with 2 and 4, form contrast training pairs. These four exercises utilize varying load intensities, with their movement velocities encompassing the majority of the force–velocity curve [8]. The fundamental essence of FCT lies in the integration of complex and contrast training. Thus, its underlying physiological mechanism is akin to both, relying on post-activation performance enhancement (PAPE) [9]. This mechanism enables practitioners to perform plyometric exercises at higher velocities or greater power outputs immediately following the heavy resistance stimulus, thereby yielding superior training adaptations. The resistance training intensity within FCT typically prescribes 80–90% of a one-repetition maximum (1RM) for the first exercise and 30–40% 1RM for the third exercise. This aligns with the traditional approach used to prescribe load intensity in many resistance training programs, known as percentage-based training (PBT). The primary advantages of PBT are its simplicity in measurement and ease of monitoring. However, an individual’s maximal strength is not static; an athlete’s daily 1RM can fluctuate significantly under different physiological states, with variations reaching up to approximately 18% [10]. Additionally, the load corresponding to an athlete’s peak power generation is highly individually specific; utilizing a standardized 30% 1RM load fails to account for these individual differences. Velocity-based strength training (VBT), however, effectively addresses both of these problems. Research by González-Badillo et al. (2015) confirmed that in core exercises such as the squat and bench press, there is a highly stable correlation (R^2^ > 0.95) between mean concentric velocity (MCV) and relative intensity (%1 RM) [11]. This implies that regardless of the daily fluctuations in an athlete’s absolute strength, a specific %1RM always corresponds to a relatively constant velocity. By measuring movement velocity at a specific submaximal load on a given day, practitioners can estimate their daily 1RM and adjust the training load in real-time to optimize training outcomes. Building upon this foundation, incorporating a force plate to measure real time power enables the construction of individual power load and power velocity relationships. This approach allows for the precise determination of the individualized peak power load and its corresponding movement velocity, thereby maximally aligning with the targeted load intensity requirements of French Contrast Training. Rebelo et al. conducted a study on velocity-based French Contrast Training involving 18 female youth roller skating athletes. Nine subjects in the experimental group performed VBT-FCT, while nine control subjects underwent conventional strength training. Following 6 weeks of training, the experimental group exhibited significant increases in countermovement jumps with and without arm swing, drop jump Reactive Strength Index with and without arm swing, and squat 1RM [12]. Huang et al. implemented an 8-week intervention utilizing velocity-controlled load training combined with plyometric training on 15 Taekwondo athletes. Their results indicated that the peak power of the CMJ in the experimental group increased from 2981.07 ± 970.04 to 3309.40 ± 978.33 (*p* < 0.001, ES = 1.64) [13]. Furthermore, compelling evidence from male volleyball athletes demonstrates that an 8-week velocity-based complex training intervention yields significantly greater enhancements in lower limb explosiveness and stretch shortening cycle capacity compared to traditional methods, primarily due to superior fatigue management and higher rep-to-rep movement velocities [14].

During the bowling action, cricket fast bowlers must generate massive vertical and horizontal ground reaction forces within a minimal timeframe at front foot contact. This rapid force application is crucial for transferring the forward kinetic energy accumulated during the run-up into forceful downward trunk rotation. Consequently, this athletic action demands a highly developed capacity for rapid lower limb force production. While VBT-FCT (or complex training) has been widely implemented as a physical conditioning modality across numerous sports, to the best of our knowledge, no study has yet investigated its training effects on cricket fast bowlers—a population whose bowling performance is highly dependent on lower limb explosive power and rapid force production. Applying this methodology to cricket fast bowlers to enhance their lower limb explosive power and rapid force production could improve crucial kinetic parameters during the delivery stride, such as peak ground reaction force, contact time, and impulse. These biomechanical adaptations may ultimately translate into increased ball release speed. Therefore, the primary objective of this study is to investigate the effects of VBT-optimized FCT on the lower limb explosive power and bowling performance of cricket fast bowlers. We hypothesize that VBT-optimized FCT will significantly improve the lower limb explosive power and bowling performance of fast bowlers, yielding substantially greater enhancements than traditional lower limb power training.

## 2. Materials and Methods

### 2.1. Experimental Approach to the Problem

All subjects were evenly allocated into an experimental group (EG) and a control group (CG). The EG underwent Velocity-Based French Contrast Training (VBT-FCT), whereas the CG completed traditional resistance training combined with traditional plyometric training. Throughout the intervention period, both groups maintained their regular bowling technique training and were strictly restricted from participating in any additional strength and conditioning regimens. The training intervention lasted for 8 weeks, with a frequency of two sessions per week. Pre- and post-intervention testing included assessments of lower limb explosive power (countermovement jump [CMJ] height, squat jump [SJ] height, Eccentric Utilization Ratio [EUR], and Reactive Strength Index [RSI]), bowling performance metrics (front foot contact [FFC] time, FFC peak ground reaction force [FFC-PF], FFC impulse, front knee angle at ball release [FKA], and ball release speed [BRS]) and Bulgarian split squat load–velocity profile (BSS L-V). One week prior to the formal commencement of the experiment, subjects were required to undertake two days of familiarization sessions. These sessions aimed to acquaint the participants with both the testing protocols and the movement techniques of the French Contrast Training, thereby ensuring that they could perform the tests and training according to the experimental design. A 48 h rest interval was maintained between the two familiarization days. An experienced strength and conditioning coach supervised and instructed the participants during this period. Following these practice sessions, all subjects demonstrated the ability to execute the testing procedures and training exercises with a standardized technique.

### 2.2. Participants

Twenty experienced medium-fast bowlers volunteered to participate in this study, including 18 right-handed and 2 left-handed fast bowlers. They were allocated to either the experimental group (EG) or the control group (CG) through a procedure where, following the completion of pre-intervention testing, they were ranked from 1 to 20 based on ball release speed from fastest to slowest and subsequently paired with adjacent participants, such as 1–2 and 3–4. A coin toss was then utilized to determine the group assignment for each pair: if the coin landed heads up, the faster bowler of the pair was assigned to the EG, whereas the slower bowler was assigned to the EG if the coin landed tails up. The primary objective of this procedure was to maintain baseline consistency between the experimental and control groups, thereby eliminating potential statistical interference in the subsequent analysis.

Their specific anthropometric characteristics, including height and body mass, are detailed in the Table 1:

Inclusion Criteria: To be eligible for participation in this study, subjects were required to meet the following criteria: (1) Active cricket player status; (2) Fast bowler primary playing position; (3) Completion of a minimum of 6 years of technical training and 4 years of resistance training; (4) Relative squat strength of 1.5 to 2.0 times their own body mass; (5) No history of cardiovascular diseases (e.g., heart disease, hypertension), tuberculosis, or other medical conditions that could impede participation in sports training; (6) No history of bone fractures or surgical procedures within the past year; (7) No history of severe musculoskeletal injuries or joint pain within the past 6 months; (8) No movement disorders or motor impairments within the past 3 months.

Exclusion Criteria: Participants were excluded if they met any of the following conditions: (1) Concurrent participation in any other systematic strength or power training programs outside of the study protocol; (2) Current use of ergogenic aids (e.g., creatine, beta-alanine) or medications known to affect neuromuscular function or hormonal status within the past three months; (3) Presence of chronic conditions that may affect balance or high-intensity exercise tolerance, such as vestibular disorders or exercise-induced asthma; (4) Anticipated inability to complete the 8-week intervention due to upcoming major competitions, travel, or other personal commitments; (5) Unwillingness to maintain their usual dietary habits and lifestyle throughout the duration of the study.

The participants ultimately included in this study possessed several years of specialized cricket training experience, with a consistent training frequency of at least 4–5 sessions per week and a duration ranging from 90 to 150 min per session. All subjects were actively competing at the national level. According to the Participant Classification Framework proposed by McKay et al. (2022), these athletes were categorized as Tier 3 (Highly Trained/National Level) fast bowlers, exhibiting a high degree of homogeneity in both technical proficiency and strength levels [15].

The study protocol was approved by the Ethics Committee of Beijing Sport University (No. 2025540H). All procedures were performed in accordance with the ethical standards of the institutional and national research committee and with the 1964 Helsinki Declaration and its later amendments. Prior to the study, all healthy adult volunteers were fully informed of the research objectives and potential risks, and they provided written informed consent.

The study was retrospectively registered on the Open Science Framework (OSF) on 22 April 2026 (https://doi.org/10.17605/OSF.IO/WE56T). The authors confirm that the trial protocol was established prior to data collection and that the retrospective registration was conducted to comply with the ICMJE guidelines for randomized controlled trials.

### 2.3. Testing Procedures

One week before and one week after the intervention, all participants completed comprehensive testing sessions. The testing protocol was conducted over a total of four days, beginning with the Bulgarian split squat load—velocity profiling across the first and second days. Subsequently, lower limb explosive power was evaluated on the third day, followed by bowling performance tests on the fourth day. To ensure adequate recovery, a 48 h interval was strictly maintained between each testing session, with all assessments successfully completed within a single week.

Test session 1: Bulgarian Split Squat Load–Velocity Profiling

Prior to the formal L-V profiling, a BSS 1-repetition maximum (1RM) test was conducted. The specific protocol was as follows: participants first completed a standardized warm-up consisting of a 10 min jog at a pace of 8 km/h, 10 min of dynamic stretching, and 5 min of hip mobility exercises. Following the warm-up, subjects performed 1 set of 10 repetitions of the BSS using an empty barbell. Subsequently, they performed 5 repetitions at 50% of their estimated 1RM, followed by 3 repetitions at 70% of their estimated 1RM, and finally, 1 repetition at 90% of their estimated 1RM. If the subject successfully completed the repetition with proper technique, the load was increased by 5%, and the attempt was repeated. This process continued until the subject failed to complete a single repetition with standard form. The load of the last successful attempt was recorded as their absolute 1RM. This maximum load was required to be determined within 3 to 5 maximal attempts.

After a 48 h recovery period, subjects returned to the testing facility for the BSS L-V profile test. The specific protocol was as follows: subjects performed 3 repetitions of the BSS at loads corresponding to 20%, 40%, and 60% of their tested 1RM, and 1 repetition at loads of 80% and 90% 1RM, respectively. The mean concentric velocity (MCV) of the barbell was recorded using a Gymaware linear position transducer (Kinetic Performance Technologies, Canberra, Australia), the reliability and validity of which have been well-established by Weakley [16]. For loads requiring 3 repetitions, the repetition with the fastest MCV was recorded for analysis. For loads requiring a single repetition, the MCV of that attempt was recorded.

The velocity data for all subjects were exported to Microsoft Excel (Version 2021; Microsoft Corp., Redmond, WA, USA). Individualized L-V profiles were established using linear regression equations. This testing protocol adhered to the methodological recommendations for constructing individualized L-V profiles proposed by Weakley [17].

Test session 2: Four Explosive Power Tests

The four explosive power tests included: CMJ height, SJ height, EUR, and RSI. Data collection was performed using a ForceDecks dual force plate system (VALD Performance, Brisbane, Australia) operating at a sampling frequency of 1000 Hz. The reliability of this system has been previously validated [18]. Following the tests on the force plates, raw kinetic data were processed via the accompanying ForceDecks software (Version 3.0.1; VALD Performance) to automatically identify movement phases and extract key kinetic variables.

For the CMJ and SJ height tests, no arm swing was allowed (hands were kept on the hips). During the CMJ height test, subjects were first weighed on the force plates and then initiated the jump following their free will. Three trials were performed, and the best performance was retained for analysis. For the SJ height test, subjects were instructed to squat down to a knee angle of 90 degrees, hold the position for two seconds, and jump vertically upward without any preparatory downward countermovement. Three trials were performed, and the best performance was retained. EUR was not directly tested but calculated using the following formula: maximum CMJ height/maximum SJ height. RSI was evaluated using the 10/5 repeated jump test. The testing protocol was as follows: the subject stood on the force plates and began upon the researcher’s signal. The subject was required to perform 10 continuous, uninterrupted vertical jumps, attempting to maximize jump height while minimizing ground contact time. The average of the 5 best jumps out of the 10 was calculated and recorded. Three sets were performed, and the best average score was retained. The RSI was calculated as: jump height/ground contact time [19].

Test session 3: Five Bowling Performance Tests

The five bowling performance tests included BRS, FFC-PF, FFC time, FFC Impulse, and FKA. FFC-PF and FFC Impulse were measured using the ForceDecks (VALD Performance, Brisbane, Australia). They were positioned in front of the bowling crease and recessed into the ground, with the top of the platforms kept flush with the surface. A custom test profile was selected in the accompanying software (Version 3.0.1; VALD Performance) to extract and analyze the continuous kinetic data from the instant of FFC during the delivery stride to the moment of ball release.

FKA and FFC time were captured using a smartphone (iPhone 16 Pro Max; Apple Inc., Cupertino, CA, USA) mounted on a tripod. The video recording resolution was set to 1080p with a sampling frequency of 240 frames per second (fps). The accuracy of utilizing smartphones for two-dimensional planar joint angle acquisition has been previously validated, and the equipment used in this study meets the requirements for general technical movement video analysis [20]. The camera’s optical axis was level with the height of the participant’s lead knee joint during the delivery stride and positioned on the same side as the lead leg (the left side of the body for right-handed bowlers and the right side for left-handed bowlers). To eliminate perspective errors, the camera was positioned 10 m away from the subject’s sagittal plane, utilizing the device’s built-in telephoto lens. To prevent data loss caused by motion blur and autofocus, the shutter speed was manually locked at 1/1000 s via a third-party professional camera application, with global exposure and focus locked simultaneously. The FFC time was defined as the duration from the initial contact of the participant’s front foot with the ground to the first visible frame of ball release in the video, representing the time elapsed from front foot contact to the instant of release. The FKA was defined as the angle of the knee joint at the exact moment the first frame of ball release was observed. Subjects wore dark athletic trousers, and bright yellow retro-reflective markers were attached to the mid-thigh, lateral epicondyle of the femur, and lateral malleolus. Subsequent video data were imported into Kinovea (2025.2.0) software for 2D coordinate calibration and joint angle extraction.

**Figure 1:**
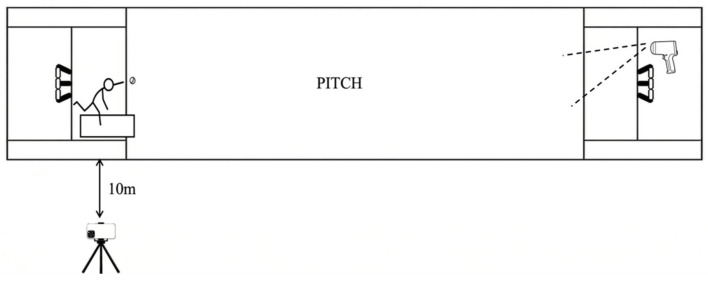
Experimental setup and equipment layout for synchronized data acquisition of bowling performance metrics.

BRS was measured using a Pocket Radar device (Smart Coach; Pocket Radar Inc., Santa Rosa, CA, USA). During the delivery, the radar gun was positioned directly facing the bowler and aligned horizontally with the height of the ball at the moment of release, to accurately capture the ball release speed. Participants performed a total of 2 overs (12 deliveries) and were instructed to perform every delivery with maximal effort, with a 2 min rest interval maintained between trials. If the ball release speed for a single delivery dropped by more than 5% relative to the previous one, an additional 5 min recovery period was provided. An experienced fast bowling coach supervised the quality of each delivery; should the quality fail to meet the required standards, a 3 min rest period was mandated, and the trial was excluded from analysis. The delivery with the highest BRS was selected for analysis. All bowling performance metrics were collected simultaneously and derived from this single fastest delivery trial. The specific experimental setup and equipment placement are illustrated in Figure 1:

### 2.4. Training Protocol

Following the pre-intervention testing sessions, both the EG and CG underwent an 8-week training program, consisting of two sessions per week. A minimum recovery period of 72 h was mandated between sessions to prevent fatigue accumulation. All training sessions were continuously monitored and supervised by at least one experienced researcher to ensure proper exercise technique. The detailed training protocols for both groups are presented in Table 2 and Table 3.

Briefly, because the primary force production pattern during the fast bowling action involves cyclical, alternating unilateral force application, unilateral exercises were selected for the training intervention—specifically, the Bulgarian Split Squat (BSS). This exercise has been demonstrated to effectively enhance athletes’ unilateral force production capabilities [21]. Furthermore, unilateral French Contrast Training (FCT) is as effective as bilateral FCT in augmenting maximal lower limb strength and explosive power; therefore, this study employed a unilateral FCT protocol based on the BSS [22].

Regarding training load prescription, the EG utilized Velocity-Based Training (VBT), setting load intensities by monitoring barbell velocity. The specific autoregulation procedures were as follows: For the maximal strength training component, the target load intensity was dictated by the specific barbell velocity corresponding to 85% 1RM, as derived from the previously conducted BSS load–velocity (L-V) profile test. Specifically, a load auto-regulation protocol was implemented before each session using the Gymaware linear position transducer. Subjects performed a movement velocity assessment during the BSS; if the mean concentric velocity deviated by more than ±0.05 m/s from the baseline velocity established at 85% 1RM, the barbell load was systematically adjusted. This ensured that the training intensity aligned with the target velocity zone prior to commencing formal sets. Additionally, a 20% velocity loss threshold was implemented for the maximal strength training component. Specifically, a set was terminated when the barbell velocity dropped by 20% compared to the velocity of the initial repetition. If the velocity of the first repetition in the subsequent set deviated by ±0.06 m/s from the target velocity of the previous set, the subject was provided with an additional 30 s of inter-set rest, and the barbell load was adjusted by 5% 1RM based on the velocity feedback until it returned to the target zone. This auto-regulation protocol was proposed and validated by Weakley et al. and is widely adopted as an effective velocity-loss training methodology [14]. For the peak power resisted plyometrics component, the target load intensity was determined by establishing individualized power-load relationships. These profiles were derived from the baseline BSS load–velocity profile captured via the ForceDecks. The specific load corresponding to the peak power output for each participant was identified and subsequently prescribed as the target training intensity. Finally, the bodyweight plyometric and assisted plyometric components remained consistent with traditional FCT protocols, with the deloaded training utilizing resistance bands to reduce the subject’s body weight by 30%, which was widely adopted by previous research [23].

The CG employed the exact same exercises and repetition volumes as the EG, but differed significantly in load prescription and overall training organization. Firstly, the CG followed a traditional straight-set resistance training sequence rather than the FCT format, completing all sets of maximal strength training, followed sequentially by weighted plyometrics, bodyweight plyometrics, and assisted plyometrics. Secondly, the CG utilized fixed relative loads, specifically 85% 1RM for maximal strength training and 30% 1RM for the weighted plyometric exercises.

At the midpoint of the intervention (after 4 weeks), all subjects underwent a re-assessment of their BSS 1RM and load–velocity profile. These data were utilized to update and prescribe the training load intensities for the subsequent four-week period.

This study established a 90% attendance requirement, necessitating the completion of at least 14 out of the 16 total training sessions for a participant to be included in the final testing. Those who failed to meet this attendance threshold were subsequently excluded from the final analysis.

### 2.5. Statistical Analysis

Experimental data were collected and organized using Microsoft Excel (Version 2021; Microsoft Corp., Redmond, WA, USA) and statistically analyzed using SPSS software (Version 27.0; IBM Corp., Armonk, NY, USA). All test data are presented as mean ± standard deviation (SD). Following the pre-intervention testing and group allocation, the Intraclass Correlation Coefficient (ICC) and Coefficient of Variation (CV) were calculated for all data with a 95% confidence interval to assess test-retest reliability. Data points with an ICC of less than 0.75 or a CV greater than 10% were excluded from the analysis. Subsequently, the Shapiro–Wilk test was employed to verify the normality of the data distribution. An independent samples *t*-test was utilized to assess whether there were significant baseline differences between the groups. An Analysis of Covariance (ANCOVA) was conducted to evaluate the significance of between-group differences in the observed changes. To control for the inflation of Type I error rate due to multiple comparisons, Bonferroni adjustments were applied to all post hoc pairwise comparisons. Statistical significance was reported using the *p*-value. When ANCOVA demonstrated statistical significance, paired samples *t*-tests were utilized to evaluate within-group changes between pre- and post-intervention testing. Effect sizes for the *t*-tests were reported using Cohen’s d based on the pre-test standard deviation (SMD_pre_), and for ANCOVA, they were reported using $\eta_{p}^{2}$. Following the computation of the effect sizes from the ANCOVA, a post hoc power analysis was executed using G*power (Version 3.1.9.7; Heinrich Heine University Dusseldorf, Dusseldorf, Germany) to evaluate the statistical sensitivity of the experimental design. This analysis utilized the observed effect sizes, an alpha level set at 0.05, and the total sample size of 20 to ascertain the achieved statistical power. Furthermore, Pearson correlation analysis was conducted to determine the relationships between BRS and other metrics that demonstrated statistical significance. The correlation coefficient (*r*) was used to report the magnitude of these relationships. In sports science, the magnitude of the effect size can be influenced by various factors, including specific performance metrics, variances in subjects’ athletic levels, and testing methodologies. According to the criteria established by Hopkins et al. (2009) [24], the thresholds for effect size (ES) are generally defined as follows: trivial (ES < 0.20), small (0.20 < ES < 0.60), moderate (0.60 < ES < 1.20), large (1.20 < ES < 2.0), and very large (ES > 2.0). This study adopted these widely recognized ES thresholds.

## 3. Results

The Shapiro–Wilk test was employed to assess the normality of the data distribution for all variables. As detailed in Appendix A Table A1, all baseline data met the assumption of normality. Independent samples *t*-tests conducted on all pre-test data revealed no significant differences between the experimental group (EG) and the control group (CG) across any metrics, with the sole exception of FFC Impulse (EG: 215.92 ± 25.19; CG: 239.31 ± 29.93; t (18) = −2.219, *p* = 0.047). Detailed statistical results are provided in Appendix A Table A2. Furthermore, to satisfy the assumptions for the paired samples *t*-test, the pre-to-post-intervention difference scores for both the EG and CG were evaluated for normality. As shown in Appendix A Table A3, except for the FFC Impulse in the EG, all difference scores exhibited a normal distribution, thereby justifying the use of the paired samples *t*-test. Consequently, the Wilcoxon signed-rank test was utilized to determine the significance of the within-group changes for the EG’s FFC Impulse.

The calculated Intraclass Correlation Coefficients (ICCs) and Coefficients of Variation (CVs) for all metrics are presented in Table 4. All variables satisfied the predetermined reliability thresholds; consequently, all data points were retained for subsequent analysis.

### 3.1. Between-Group Analysis: ANCOVA Results

The results of the Analysis of Covariance (ANCOVA) represent the core findings of this study. Prior to conducting the ANCOVA, a fundamental assumption—the homogeneity of regression slopes—was evaluated. This was achieved by examining the interaction effect between the pre-test covariate and the group assignment (Pre-test × Group) for all variables. The analysis revealed no significant interaction effects across any of the datasets (*p* > 0.05), thereby successfully satisfying this prerequisite assumption. Consequently, the ANCOVA was robustly executed to compare the post-intervention differences between the groups. The detailed results are presented below. Furthermore, the results of post hoc power analysis for all metrics are presented in Table 4, reported as 1-*β* err prob.

Post hoc power analysis revealed that, given a total sample size of 20 and an alpha level set at 0.05, the primary outcome measure (BRS) achieved a statistical power of 0.99. Furthermore, the statistical power for all variables exhibiting significant differences exceeded 0.75. These findings indicate that the sample size of 20 participants was adequate to satisfy the experimental design requirements of the present study.

Based on the statistical analysis outcomes, the experimental group (EG) demonstrated a significantly greater improvement in CMJ height compared to the control group (CG) (*p* < 0.001, ES = 0.659). However, no significant between-group difference was observed for SJ height (*p* = 0.659). Furthermore, the EG exhibited significantly superior enhancements in both the Eccentric Utilization Ratio (EUR) (*p* = 0.019, $\eta_{p}^{2}$ = 0.281) and Reactive Strength Index (RSI) (*p* = 0.004, $\eta_{p}^{2}$ = 0.399) when compared to the CG. Regarding the bowling performance metrics, the ANCOVA revealed no significant between-group differences for FFC time and FFC-PF (*p* = 0.125, 0.117). Conversely, the EG achieved significantly greater increases in FFC Impulse (*p* < 0.001, $\eta_{p}^{2}$ = 0.572), FKA (*p* < 0.001, $\eta_{p}^{2}$ = 0.590), and BRS (*p* < 0.001, $\eta_{p}^{2}$ = 0.704) compared to the CG.

Analysis of the Bulgarian split squat (BSS) load–velocity relationship indicated that the EG achieved significantly superior velocity adaptations within the 20% to 60% 1RM range (*p* = 0.008, 0.011, 0.008, $\eta_{p}^{2}$ = 0.327, 0.313, 0.324). In contrast, no significant between-group differences in movement velocity were detected in the heavier 80% and 90% 1RM zones (*p* = 0.094, 0.074).

These findings indicate that the VBT-FCT protocol yielded significantly greater improvements in lower limb power and movement velocity under low or zero resistance conditions compared to traditional training. This superior adaptation ultimately resulted in a significantly larger increase in ball release speed for the experimental group relative to the control group.

### 3.2. Within-Group Analysis: Experimental Group

Pre- and post-intervention data for the experimental group were aggregated and analyzed using a paired samples *t*-test. The results are summarized as follows (Table 5):

As the pre-to-post intervention difference scores for FFC Impulse in the EG violated the assumption of normality, a Wilcoxon signed-rank test was employed to determine statistical significance. The results are presented in Table 6:

According to Table 5 and Table 6, following the 8-week Velocity-Based French Contrast Training (VBT-FCT) intervention, the experimental group (EG) demonstrated significant improvements across all metrics. Notably, CMJ height exhibited the most substantial enhancement (ES = 2.864), yielding a mean pre-to-post-intervention difference of 3.7 cm. Furthermore, all other variables—except RSI and FFC Impulse—produced large effect sizes (ES > 1.2). Although the effect size for FFC Impulse could not be directly calculated, the resulting *p* value indicates a statistically significant increase compared to the baseline assessment (*p* = 0.005). Specifically, BRS yielded a large effect size (ES = 1.767) with a mean increase of 2.08 m/s, which represents a highly meaningful and practically significant improvement for fast bowlers. Regarding the Bulgarian split squat (BSS) movement velocities across different load intensities, the EG achieved significant velocity increases within the 20% to 60% 1RM range (*p* < 0.001, = 0.003, = 0.006), demonstrating moderate-to-large effect sizes (ES = 0.853, 1.838, 1.807).

Consequently, following the 8-week intervention, the experimental group exhibited significant enhancements in lower limb explosive power across both slow and fast velocities, alongside increased concentric movement speed under low resistance. These physiological adaptations positively transferred to the bowling mechanics, manifesting as increased front foot contact impulse and improved knee joint impact tolerance, ultimately driving the observed improvements in ball release speed.

### 3.3. Within-Group Analysis: Control Group

Pre- and post-intervention data for the control group (CG) were aggregated and analyzed using a paired samples *t*-test. The results are summarized as follows (Table 7):

According to Table 7, following the 8-week traditional resistance and plyometric training intervention, the control group (CG) demonstrated significant improvements in CMJ and SJ heights (*p* < 0.001), exhibiting moderate effect sizes (ES = 0.663, 0.664). The Eccentric Utilization Ratio (EUR) showed no significant improvement from pre-test (*p* = 0.243, ES = 0.664). The Reactive Strength Index (RSI) improved significantly (*p* = 0.007), although the effect size was relatively small (ES = 0.122). For bowling performance metrics, no significant improvements were observed in FFC Impulse (*p* = 0.243), FKA (*p* = 0.113) and BRS (*p* = 0.188). Regarding the BSS load–velocity profile, the CG exhibited significant velocity enhancements specifically at 20% and 40% 1RM (*p* = 0.042, 0.011), with small and medium effect size (ES = 0.338, 1.143). No significant improvement was observed from 60% 1RM L-V (*p* = 0.065, ES = 0.613).

It is evident that following the 8-week intervention of combined resistance and plyometric training, the control group demonstrated a significant improvement in lower limb explosive power. However, their capacity to utilize the stretch shortening cycle did not exhibit a substantial increase, as indicated by a lack of statistical significance for EUR and only a small effect size for RSI. Movement velocity under low resistance conditions showed marginal growth, yielding only small or moderate effect sizes. Furthermore, the acquired gains in lower limb power failed to transfer into bowling performance, with no significant improvements observed in the various lower limb kinetic variables or the primary outcome measure BRS.

### 3.4. Correlation Analysis: BRS with Other Metrics

A correlation analysis was conducted between the change scores of all variables demonstrating significant differences in the main effect analysis and the change score of the primary outcome measure, ball release speed. The results are as follows (Table 8):

According to Table 8, statistical results reveal that the pre- and post-intervention difference in FFC impulse had the strongest correlation with the change in BRS (*p* < 0.001, *r* = 0.906). All other variables, excluding the 60% 1RM L-V, displayed moderate correlations, while the 60% 1RM L-V difference was not significantly correlated with BRS (*p* = 0.054, *r* = 0.436). These findings highlight that BRS variations are intimately linked to lower limb power. Most notably, the vertical ground reaction impulse generated by the front foot during the delivery phase plays a decisive role in determining ball speed. Because the experimental cohort achieved significantly greater enhancements in this exact kinetic parameter than the control group, they consequently attained a highly significant increase in BRS.

### 3.5. Force–Time Curve for Front Foot Contact

**Figure 2:**
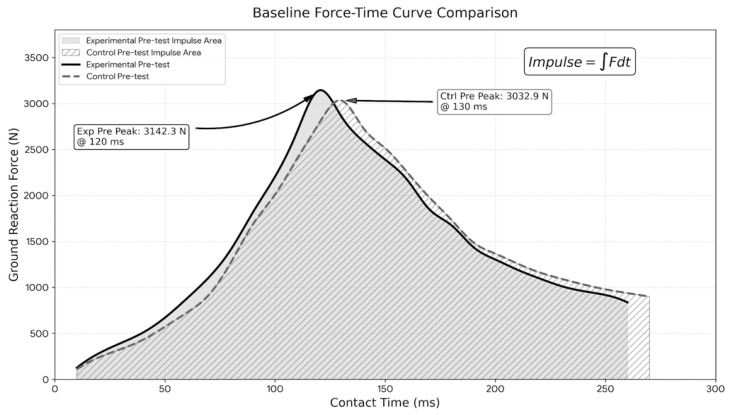
Front foot contact impulse of EG and CG at pre-test.

**Figure 3:**
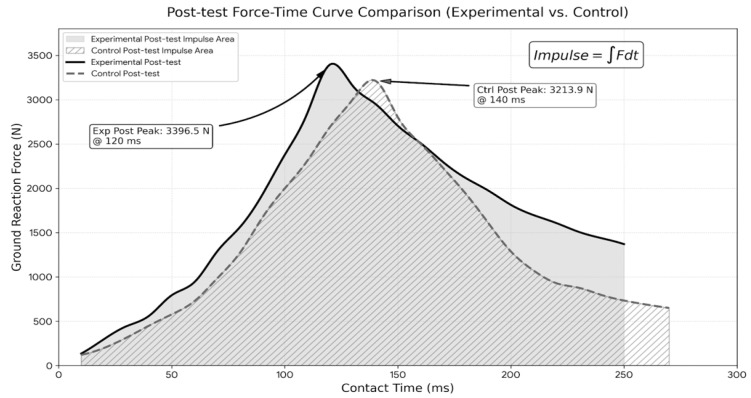
Front foot contact impulse of EG and CG at post-test.

Figure 2 and Figure 3 illustrate the comparison of force–time curves for the experimental and control groups during the bowling delivery from the moment of front foot contact to ball release. The area under the curve shown in these graphs represents the FFC Impulse during the pre-test and post-test assessments.

Based on the preceding statistical results, both the experimental and control groups exhibited varying degrees of improvement in FFC PF following the intervention, although these increases did not reach statistical significance (*p* = 0.117, $\eta_{p}^{2}$= 0.138). Conversely, the experimental group achieved a significantly greater enhancement in FFC Impulse compared to the control group (*p* = 0.001, $\eta_{p}^{2}$ = 0.572). When examining the accompanying figures, it becomes apparent that the experimental group attained peak force earlier than the control group. Furthermore, the decline in force during the latter half of the curve was considerably more gradual, indicating that the overall gain in impulse was primarily derived from this extended phase of force application.

### 3.6. Bulgarian Split Squat Load–Velocity Profile

**Figure 4:**
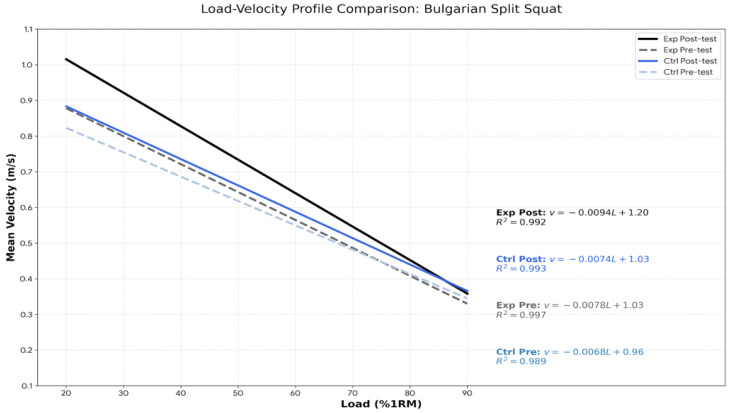
Pre- and Post-Bulgarian split squat load–velocity profiles for experimental and control groups.

In addition to the athletic performance tests, this study established an individualized load–velocity (L-V) profile for each subject. This profiling served a dual purpose: first, to inform the individualized load prescription for the experimental group (EG); and second, to quantify the post-training adaptations in movement velocity across a complete spectrum of relative loads. Figure 4 illustrates the pre-to-post-intervention comparisons of the Bulgarian split squat (BSS) L-V curves for both the EG and the CG:

Combining the statistical results with the visual figures, it is evident that following the 8-week intervention, the experimental group achieved a significant increase in mean concentric velocity under low resistance (<60% 1RM) and exhibited a significant difference compared to the control group (*p* < 0.05). The control group experienced some improvement in mean concentric velocity under very low resistance (<40% 1RM), but the effect size was small and lacked practical significance (ES = 0.338, 1.143). As the load increased, both groups demonstrated a certain degree of growth in mean concentric velocity under high resistance conditions, yet the between-group comparison revealed no significant difference (*p* > 0.05). This implies that velocity-based French Contrast Training can effectively enhance subject movement velocity under low resistance, whereas its improvement on movement velocity under high resistance does not possess a significant advantage over traditional resistance training combined with plyometric training.

## 4. Discussion

The results demonstrated that compared to the control group (CG), the experimental group (EG) exhibited significantly greater improvements in CMJ Height, EUR, RSI, FFC Impulse, FKA, and BRS. Conversely, no significant between-group differences were observed for SJ Height, FFC Time, and FFC PF. For BSS L-V Profile, EG showed significant improvement of moving velocity under load 20% 1RM to 60% 1RM compared to CG. No significant difference of moving velocity observed under 80% 1RM and 90% 1RM load intensity. These findings indicate that VBT-FCT, when compared to traditional lower limb power training, is indeed a more effective training modality for enhancing the lower limb explosive power and athletic performance of fast bowlers, which is largely consistent with our initial hypothesis.

### 4.1. Lower-Limb Kinetics Performance

French Contrast Training effectively enhances the lower limb explosive power of subjects, a principle largely attributed to its utilization of Post Activation Potentiation and Post Activation Performance Enhancement [25,26]. During training, FCT enables subjects to execute plyometric exercises at a higher intensity following resistance training, thereby increasing the overall training stimulus and amplifying the training adaptations. While retaining the individual physiological characteristics of both training modalities, FCT further intensifies the impact of these potentiating effects, prolonging their duration and allowing athletes to sustain higher power outputs over an extended period [27].

In conjunction with our statistical results, the experimental group demonstrated a significant advantage in the improvement in CMJ height compared to the control group, whereas no such significant advantage was observed for SJ height. This indicates that FCT enhanced the ability of the subjects to utilize the stretch shortening cycle (SSC) [28,29]. However, for isolated concentric explosive power, FCT did not elicit superior training effects. Similar outcomes are reflected in the EUR and RSI metrics. EUR represents the ratio of CMJ height to SJ height; the substantial increase in CMJ for the experimental group inevitably drives the elevation of EUR, serving as crucial evidence for the effective reinforcement of their SSC [30]. RSI, the ratio of flight time to contact time, places a greater emphasis on the ability of the subject to utilize the fast stretch shortening cycle (FSSC) compared to EUR, as the contact time is typically less than 250 ms [31]. The significant improvement in RSI proves that FCT can effectively enhance tendon elasticity and joint stiffness, thereby improving the capacity of the subjects to utilize the fast stretch shortening cycle.

Within the back squat load velocity profile, the experimental subjects achieved a significant increase in mean concentric velocity under low resistance conditions. However, as the load intensity increased, the advantage of the experimental group over the control group gradually diminished until it vanished. When Rebelo et al. implemented velocity-based FCT to train youth female roller skaters, they observed a significant increase in both CMJ and drop jump heights for the experimental group, but no significant improvement in the mean concentric velocity of squats and hip thrusts under high resistance, which is consistent with the findings of the present study [12]. Furthermore, in a meta-analysis of previously published FCT literature, Zhao et al. discovered that while FCT exerts a significantly better effect on explosive power development, its impact on maximal strength or the rate of force production under high resistance is comparable to that of traditional training [23]. These collective results suggest that FCT effectively enhances the ability to utilize the stretch shortening cycle, an improvement potentially mediated by increased tendon elasticity and joint stiffness. Nevertheless, it does not demonstrate a clear advantage for maximal strength during concentric muscle contractions.

The enhancement of dynamic explosive power is primarily driven by the full utilization of PAPE. Research indicates that high-intensity contractions lead to a significant elevation in muscle temperature, accelerate ATPase activity, increase neural conduction velocity, and reduce muscle and joint viscosity. These factors form a vital foundation for the improved performance of FCT movements. Additionally, following heavy resistance training, the short-term increase in motor unit recruitment—specifically, high-threshold type II motor units, the elevation in motor neuron excitability, and the reduction in presynaptic inhibition characterized by an enhanced H reflex—allows subjects to perform subsequent training at a higher intensity, yielding superior training adaptations [26]. Performing plyometric training over extended periods in this potentiated state can significantly enhance muscle and tendon elasticity, as well as joint stiffness. Moreover, the recruitment of high-threshold motor units can induce specific stretch shortening cycle adaptations. This optimizes the regulation of agonist and antagonist muscles by the nervous system, allowing athletes to achieve more efficient energy storage and release during the instantaneous transition from the eccentric to the concentric phase [32].

Although FCT effectively augments performance in dynamic movements, its capacity to improve maximal strength and static explosive power remains limited. The enhancement of these two qualities heavily relies on increased muscle cross sectional area and extreme improvements in maximal absolute strength, which in turn are highly dependent on the muscle sustaining a sufficient time under tension under extremely high loads. Among the four sequential exercises in FCT, only one focuses on heavy resistance. Consequently, the total volume load dedicated specifically to maximal strength is substantially lower than that of traditional pure strength training modules. Furthermore, the fundamental nature of FCT is a high intensity complex circuit. After completing the initial heavy load exercise, the subsequent three high speed movements rapidly deplete local adenosine triphosphate and phosphocreatine reserves, while simultaneously triggering central nervous system fatigue. This fatigue cannot be completely dissipated between sets, which directly inhibits the ability of the central nervous system to output maximal neural drive during the heavy squats of the following set. As a result, subjects are unable to maintain the true load intensity required to sufficiently stimulate maximal absolute strength throughout the entire training session [12,23,33]. During an FCT intervention involving collegiate badminton players, Zhou et al. discovered that the pre- and post-intervention changes in the high resistance zone from 60% to 80% 1RM showed no significant improvement compared to the control group, which aligns fundamentally with the results of our study [33].

In addition to the application of the French Contrast Method, the present study incorporated velocity-based training to assist in autoregulating load intensity within the contrast complex. When Rebelo et al. implemented velocity-based French Contrast Training for youth female roller skaters, they prescribed a 20% velocity loss threshold for both heavy back squats and hip thrusts. Their results indicated a significant enhancement in explosive power for the experimental group. Furthermore, in an investigation examining the effects of velocity-based complex training on the lower limb maximal strength and power of volleyball athletes, Lin et al. utilized a target velocity corresponding to 85% 1RM coupled with a 10% velocity loss threshold for the experimental cohort. Their findings revealed that the countermovement jump height and eccentric utilization rate of the experimental group were significantly superior to those of the control group, whereas squat jump height and back squat 1RM demonstrated no significant advantage. Pareja Blanco et al. investigated the impact of various velocity loss thresholds on strength and power adaptations. They discovered that a velocity loss of between 10% and 20% optimally promotes the rate of force development while preserving absolute strength gains. Conversely, a velocity loss of 40% or greater, although effective for increasing muscle cross sectional area, ultimately impairs the development of explosive power [12,14,34]. The current study similarly adopted the movement velocity corresponding to 85% 1RM alongside a 20% velocity loss threshold, yielding results consistent with the previous literature. Specifically, substituting prescribed static weights and fixed repetitions with target velocities and precise velocity loss thresholds yields significantly greater enhancements in lower limb explosive power.

### 4.2. Bowling Performance

During the delivery stride, cricket fast bowlers are highly dependent on the braking force exerted by the lower limbs against the ground to convert forward kinetic energy into the rotational kinetic energy of the trunk, ultimately propelling the ball [35,36]. Biomechanical investigations of cricket fast bowling by Hurrion et al. (2000) demonstrated that at the instant of delivery, the peak ground reaction forces transferred by fast bowlers can reach up to 6 times body weight (6 BW) in the vertical direction and 4 BW in the horizontal direction [37]. Furthermore, Portus et al. (2006), in a study of 42 elite Australian male fast bowlers, concluded that higher ball speeds are associated with longer front foot contact times and greater braking and vertical ground impulses [38]. Consequently, for fast bowlers to achieve higher delivery speeds, the front foot must generate greater force and impulse against the ground during the delivery stride, a principle that aligns fundamentally with the findings of the present study.

However, a notable divergence exists. In this study, the experimental group (EG) exhibited a significantly greater increase in ball release speed (BRS) compared to the control group (CG); yet, the enhancement in their peak ground reaction force at front foot contact (FFC-PF) did not differ significantly from that of the CG, indicating that changes in peak force do not inherently dictate the final ball velocity. This specific finding conflicts with some of the aforementioned literature [36,37]. To elucidate this discrepancy, the present study extracted the force–time (F-t) curves of the subjects during the bowling trials, capturing the continuous kinetic data from the instant the front foot contacted the force plate to the moment of ball release.

As illustrated in the force–time graphs, following the 8-week intervention, both the experimental group (EG) and the control group (CG) exhibited varying degrees of increase in FFC-PF (EG: 227.2 N; CG: 181.0 N). However, the ANCOVA results revealed no significant between-group difference in the magnitude of this increase (*p* = 0.117, $\eta_{p}^{2}$ = 0.138). By examining the shaded areas under the curves in conjunction with the pre-intervention independent samples *t*-test, EG had a significantly lower baseline FFC Impulse compared to the CG (detailed in Appendix A Table A2). After the 8-week training period, this metric improved significantly in the EG, with the magnitude of enhancement vastly exceeding that of the CG (*p* < 0.001, $\eta_{p}^{2}$ = 0.572). Furthermore, the correlation analysis presented in the results section revealed that the change in FFC Impulse is highly correlated with the change in ball release speed (*p* < 0.001, *r* = 0.906). Therefore, the critical determinant strongly correlated with fast bowling speed is the impulse generated against the ground following front foot contact, rather than the instantaneous peak force. King et al. collected kinematic and kinetic bowling data from twenty elite male fast bowlers. Their analysis revealed a moderate but non-significant correlation between ball speed and peak vertical ground reaction force, indicating that faster bowlers tended to generate lower peak vertical ground reaction forces (*r* = −0.364, *p* = 0.114). Additionally, a greater horizontal braking impulse was significantly associated with a faster ball release speed (*r* = 0.574, *p* = 0.008). This study is fundamentally consistent with the findings of the present research, as it links ball velocity to impulse rather than peak force [39].

According to Figure 2 and Figure 3, following the 8-week intervention, the experimental group demonstrated a shorter time to peak force during the front foot contact phase of the delivery stride, specifically in the initial segment of the curve. This indicates that the experimental group possessed a higher rate of force development (RFD), imparting a greater impulse to the ground within a shorter timeframe. In contrast, while the traditional resistance and plyometric training undertaken by the CG also improved general explosive power—thereby increasing peak force—the improvement in RFD and impulse did not show any significance. Consequently, it is evident that French Contrast Training significantly enhances the capacity to rapidly generate force within a minimal timeframe. This is achieved by inducing post activation performance enhancement, enabling subjects to execute high-intensity plyometrics in a potentiated state. Furthermore, implementing a unilateral movement template for the French Contrast complex, specifically, the Bulgarian split squat, further elevated their unilateral force expression capabilities, which ultimately translated into improved bowling performance.

In addition to RFD, the correlation analysis revealed that the front knee angle at ball release (FKA) was also highly correlated with ball release speed (BRS). Combined with the statistical results, the experimental group (EG) demonstrated a significantly greater FKA post-intervention (*p* < 0.001, $\eta_{p}^{2}$ = 0.876), and this improvement was significantly superior to that of the control group (CG) (*p* < 0.001, $\eta_{p}^{2}$ = 0.59).

During the fast bowling delivery, to successfully transfer the momentum accumulated from the run-up into trunk rotation, the front foot must actively apply braking force against the ground while withstanding tremendous impact forces. Consequently, the knee joint often undergoes passive flexion to absorb this impact. However, elite fast bowlers exhibit high knee joint stiffness and minimal flexion amplitude. Furthermore, multiple studies have indicated that a larger (more extended) knee angle corresponds to less passive absorption of kinetic energy and smoother momentum transfer, ultimately resulting in a faster BRS [36,40,41]. The findings of the present study are highly consistent with this established literature.

According to the latter half of the force–time curves in Figure 2 and Figure 3, when the front foot brakes during the delivery stride and the ground reaction force reaches its peak, the knee begins to flex to attenuate the impact. At this critical juncture, greater knee joint stiffness and superior lower limb eccentric resistance capacity result in less knee flexion. This enhanced rigidity naturally generates a larger impulse against the ground, facilitating a more seamless conversion of kinetic energy. Velocity-based French Contrast Training enhances lower limb power output by identifying the exact load that elicits peak power for each individual subject and executing the subsequent resistance plyometric training at this precise intensity. Furthermore, consistently training within this peak power output zone significantly elevates knee and ankle joint stiffness, while simultaneously enhancing the eccentric resistance capacity of the lower limb extensors. During the latter half of the force–time curve, the rate of force decay in the experimental group was significantly slower than that of the control group, thereby contributing a greater amount of impulse compared to the initial phase. Sustaining a greater ground reaction force implies less impact attenuation by the lower extremities, which consequently corresponds to a larger knee joint angle. This mechanism also elucidates the moderate correlation observed between the knee joint angle and ball release speed. This relationship is fundamentally mediated by the association of the knee angle with the front foot contact impulse, rather than a direct independent correlation with ball velocity.

Among the five bowling performance metrics, front foot contact time (FFC time) was the sole variable that exhibited no significant change in either the experimental group (EG) or the control group (CG). FFC time refers to the duration from the instant the front foot strikes the ground during the delivery stride to the exact moment of ball release. According to Worthington et al. regarding the identification of key kinematic determinants in fast bowlers, the FFC time of elite fast bowlers (ball release speed > 130 km/h) demonstrates a significant negative correlation with ball velocity (*r* = −0.602) [41]. Typically, this FFC time for elite bowlers ranges from 0.12 s to 0.20 s, varying considerably based on the bowler’s specific force production mechanics and their bowling action classification (i.e., front-on, side-on, or mixed/semi-open actions) [40,41,42].

Furthermore, during the front foot contact phase, the bowler must execute a highly complex sequence of movements: generating lower-limb force, transferring it seamlessly through the entire kinetic chain to the distal extremity, and ultimately releasing the ball. The fluidity and sequencing of this process possess a critical impact on final ball velocity. Crucially, the time required to complete this sequence depends heavily on how efficiently the body segments above the hip joint transfer the ground reaction forces generated by the lower limbs into the bowling hand. This transfer efficiency is not absolutely dictated by lower-limb strength or explosive power.

Consequently, training interventions strictly targeting lower-limb power development cannot inherently alter the temporal characteristics of this entire kinetic sequence. This biomechanical reality likely represents the primary reason for the absence of significant pre-to-post-intervention changes in FFC time among the subjects. In essence, FFC time is predominantly governed by overall technical proficiency and upper-body sequencing, rather than purely by lower-limb explosive power or speed-strength. Therefore, the two lower-limb-focused explosive training protocols implemented in this study did not effectively modify (i.e., reduce) the front foot contact time of the fast bowlers.

## 5. Practical Applications

The findings of this study offer vital guidance for strength and conditioning professionals aiming to optimize the explosive power of elite fast bowlers. Regarding training periodization, we highly recommend incorporating this velocity-based French Contrast Training protocol during the late pre-season phase. Utilizing this method twice weekly allows athletes to maximize neuromuscular adaptations and peak lower limb power output just before entering the competitive season. During the in-season phase, the training frequency could be reduced to once weekly as a potent stimulus to maintain explosive performance without accumulating residual fatigue. Furthermore, this protocol presents substantial implications for injury prevention. The strict implementation of a 20 percent velocity loss threshold serves as an objective autoregulatory tool. By terminating sets before excessive movement velocity degradation occurs, coaches can effectively limit intra-session mechanical stress and neurological fatigue. This objective load management strategy is essential for mitigating the risk of overtraining and preventing overuse injuries commonly associated with the high impact nature of fast bowling.

## 6. Conclusions

Velocity-based French Contrast Training enhances the lower limb explosive power of cricket fast bowlers, thereby improving lower limb kinetic performance during the delivery stride, and ultimately increasing ball release speed. Furthermore, this training method is more effective than traditional resistance training combined with plyometrics.

## 7. Limitations

This study has several limitations that should be acknowledged:

1. Fast bowling is a highly complex technical skill. Beyond lower limb explosive power, the technical variability of the bowler during the delivery may also influence the measurement results. The current study did not strictly control the individual bowling technique of the subjects. Consequently, future research should carefully monitor kinematic variations during the bowling process.
2. Throughout the course of this study, only the specific training protocols of the subjects encompassing both physical conditioning and technical practice were strictly regulated. Extraneous lifestyle factors unrelated to the immediate training environment, such as daily nutritional intake and rest periods, were not rigorously monitored. Future research should aim to control these confounding variables to further enhance the reliability of the experimental outcomes.
3. This study utilized a single-camera 2D video analysis system to acquire knee kinematic data. Although rigorous camera placement protocols were implemented to minimize parallax and perspective errors, the cricket fast bowling action involves substantial multi-planar rotations of the hip, lumbar spine, and thoracic spine. Consequently, out-of-plane joint movements in the frontal and transverse planes may have introduced inherent artifacts into the sagittal plane angle calculations. Future investigations should employ 3D motion capture systems for more robust kinematic validation.
4. Constrained by the inherent difficulties associated with recruiting high-level fast bowlers, the relatively small sample size (N = 20) may have limited the statistical power of the ANCOVA models. This likely constitutes a primary reason why several secondary performance metrics failed to achieve statistical significance.
5. This study exclusively evaluated ball release speed (BRS) as the ultimate criterion measure of performance. However, bowling accuracy is an equally critical determinant of a fast bowler’s overall efficacy. Future research should incorporate accuracy assessments to provide a more comprehensive evaluation of the training intervention.
6. Following the conclusion of the experiment, this study did not continuously track the changes in lower limb explosive power and bowling performance of the subjects. Consequently, it is impossible to determine the specific duration for which the training method can continuously affect athletic performance. It is highly recommended that future studies conduct follow up assessments on the participants for a designated period to establish the exact duration of the training efficacy.
7. The participant cohort in this study consisted entirely of males. The applicability and comparative efficacy of this VBT-FCT protocol for female cricket fast bowlers remain unknown and warrant further investigation.

## Figures and Tables

**Table 1:** Anthropometric characteristics of the medium-fast nowlers.

	Number	Age	Height (cm)	Weight (kg)	Years of Training
		M ± SD	M ± SD	M ± SD	M ± SD
EG	10	22.1 ± 2.33	181.96 ± 2.18	78.6 ± 2.15	6.5 ± 1.43
CG	10	22.2 ± 1.69	181.98 ± 2.11	78.6 ± 2.13	6.5 ± 0.85

**Table 2:** EG training protocol.

Movement	Intensity	Repetitions	Sets	Interval
Heavy load BBS	85% 1RM velocity ± 0.05	20% velocity loss	3	20 s between movements and 3 min between sets
Bodyweight BBS plyometrics	bodyweight	4	3	
Resisted BBS plyometrics	load at peak power	4	3	
Assisted BBS plyometrics	30% bodyweight deload	4	3	

**Table 3:** CG training protocol.

Movement	Intensity	Repetitions	Sets	Interval
Heavy load BBS	85% 1RM	4	3	1 min between sets and 3 min between movements
Resisted BBS plyometrics	30% 1RM	4	3	
Bodyweight BBS plyometrics	Bodyweight	4	3	
Assisted BBS plyometrics	30% bodyweight deload	4	3	

**Table 4:** Between-group ANCOVA analysis.

Measurement	EG (Pre)	EG (Post)	CG (Pre)	CG (Post)	*t*-Value	*p*-Value	$\eta_{p}^{2}$	ICC (95%CI)	CV (%)	1-*β*
CMJ height	45.72 ± 1.29	49.42 ± 1.06	44.69 ± 3.80	47.21 ± 3.60	5.728	<0.001	0.659	0.91 (0.86–0.93)	2.15	0.99
SJ height	43.05 ± 1.65	44.82 ± 1.84	42.53 ± 3.84	44.18 ± 4.10	0.299	0.769	0.005	0.86 (0.83–0.89)	4.34	0.06
EUR	1.06 ± 0.02	1.10 ± 0.26	1.06 ± 0.02	1.07 ± 0.01	2.581	0.019	0.281	0.88 (0.85–0.91)	3.82	0.75
RSI	3.09 ± 0.20	3.21 ± 0.16	3.22 ± 0.20	3.24 ± 0.20	3.357	0.004	0.399	0.97 (0.92–0.99)	5.86	0.92
FFC Time	0.26 ± 0.03	0.25 ± 0.01	0.27 ± 0.03	0.27 ± 0.02	−1.613	0.125	0.133	0.95 (0.94–0.98)	2.45	0.38
FFC PF	3142.20 ± 160.61	3396.4 ± 145.89	3032.90 ± 216.95	3213.90 ± 209.67	1.649	0.117	0.138	0.94 (0.91–0.98)	3.41	0.39
FFC Impulse	215.92 ± 23.90	254.90 ± 26.04	239.31 ± 23.93	244.38 ± 25.70	4.467	<0.001	0.572	0.87 (0.79–0.95)	5.06	0.99
FKA	144.61 ± 3.03	150.3 ± 3.08	144.07 ± 5.82	145.33 ± 5.37	4.950	<0.001	0.590	0.93 (0.90–0.95)	2.57	0.99
BRS	29.82 ± 1.18	31.90 ± 1.36	30.78 ± 1.19	31.01 ± 1.14	6.354	<0.001	0.704	0.93 (0.87–0.97)	3.01	0.99
20% 1RM L-V	0.87 ± 0.15	0.99 ± 0.11	0.85 ± 0.09	0.88 ± 0.11	3.194	0.008	0.327	0.96 (0.91–0.98)	2.29	0.84
40% 1RM L-V	0.73 ± 0.06	0.84 ± 0.09	0.67 ± 0.07	0.75 ± 0.06	2.861	0.011	0.313	0.94 (0.87–0.96)	2.82	0.81
60% 1RM L-V	0.56 ± 0.05	0.65 ± 0.06	0.52 ± 0.05	0.55 ± 0.03	3.146	0.008	0.324	0.88 (0.82–0.94)	3.17	0.83
80% 1RM L-V	0.42 ± 0.05	0.46 ± 0.03	0.43 ± 0.03	0.45 ± 0.02	1.838	0.094	0.175	0.86 (0.84–0.89)	3.93	0.49
90% 1RM L-V	0.32 ± 0.02	0.33 ± 0.03	0.35 ± 0.02	0.37 ± 0.03	−1.984	0.074	0.188	0.94 (0.89–0.96)	4.77	0.53

**Table 5:** EG within-group paired samples *t*-test.

Measurement	Time	Outcomes	*t*-Value	*p*-Value	SMD_pre_
CMJ height	Pre-	45.72 ± 1.29	−20.402	<0.001	−2.864
	Post-	49.42 ± 1.06			
EUR	Pre-	1.06 ± 0.02	−8.128	<0.001	−2.320
	Post-	1.10 ± 0.26			
RSI	Pre-	3.09 ± 0.20	−5.009	<0.001	−0.614
	Post-	3.21 ± 0.16			
FKA	Pre-	144.61 ± 3.03	−9.259	<0.001	−1.876
	Post-	150.3 ± 3.08			
BRS	Pre-	29.82 ± 1.18	−10.554	<0.001	−1.767
	Post-	31.90 ± 1.36			
20% 1RM L-V	Pre-	0.87 ± 0.15	−5.458	<0.001	−0.853
	Post-	0.99 ± 0.11			
40% 1RM L-V	Pre-	0.73 ± 0.06	−4.062	0.003	−1.838
	Post-	0.84 ± 0.09			
60% 1RM L-V	Pre-	0.56 ± 0.05	−3.631	0.006	−1.807
	Post-	0.65 ± 0.06			

**Table 6:** EG within-group Wilcoxon signed-rank test.

Measurement	*z*-Value	*p*-Value
FFC Impulse	−2.803 b	0.005

Note: Symbol “b” means software calculation based on positive ranks.

**Table 7:** CG within-group paired samples *t*-test.

Measurement	Time	Outcomes	*t*-Value	*p*-Value	SMD_pre_
CMJ height	Pre-	44.69 ± 3.80	−10.747	<0.001	−0.663
	Post-	47.21 ± 3.60			
EUR	Pre-	1.06 ± 0.02	−1.468	0.176	−0.664
	Post-	1.07 ± 0.01			
RSI	Pre-	3.22 ± 0.20	−3.497	0.007	−0.122
	Post-	3.24 ± 0.20			
FFC Impluse	Pre-	239.31 ± 23.93	−1.249	0.243	−0.212
	Post-	244.38 ± 25.70			
FKA	Pre-	144.07 ± 5.82	−1.754	0.113	−0.216
	Post-	145.33 ± 5.37			
BRS	Pre-	30.78 ± 1.19	−1.426	0.188	−0.193
	Post-	31.01 ± 1.14			
20% 1RM L-V	Pre-	0.85 ± 0.09	−2.535	0.032	−0.338
	Post-	0.88 ± 0.11			
40% 1RM L-V	Pre-	0.67 ± 0.07	−3.093	0.011	−1.143
	Post-	0.75 ± 0.06			
60% 1RM L-V	Pre-	0.52 ± 0.05	−2.174	0.065	−0.613
	Post-	0.55 ± 0.03			

**Table 8:** Correlation analysis for BRS with other metrics.

Measurement	Correlation (r)	*p*-Value
CMJ Height—BRS	0.563	0.010
EUR—BRS	0.578	0.008
RSI—BRS	0.588	0.006
FFC Impulse—BRS	0.906	<0.001
FKA—BRS	0.575	0.008
20% 1RM L-V—BRS	0.593	0.006
40% 1RM L-V—BRS	0.478	0.033
60% 1RM L-V—BRS	0.436	0.054

## Data Availability

The original contributions presented in the study are included in the article; further inquiries can be directed to the corresponding author.

## Appendix A

**Table A1:** Shapiro–Wilk tests of normality of the pre-intervention measurements of the explosive performance, bowling performance, and Bulgarian L-V profile.

Measurement	Group	Statistic	*df*	*p*-Value
CMJ height	EG	0.940	9	0.554
	CG	0.950	9	0.664
SJ height	EG	0.947	9	0.928
	CG	0.929	9	0.437
EUR	EG	0.939	9	0.537
	CG	0.881	9	0.136
RSI	EG	0.878	9	0.124
	CG	0.873	9	0.108
FFC time	EG	0.982	9	0.975
	CG	0.948	9	0.664
FFC PF	EG	0.908	9	0.268
	CG	0.884	9	0.146
FFC Impulse	EG	0.923	9	0.380
	CG	0.966	9	0.848
FKA	EG	0.987	9	0.992
	CG	0.950	9	0.663
BRS	EG	0.985	9	0.987
	CG	0.957	9	0.751
20% 1RM L-V	EG	0.943	9	0.715
	CG	0.857	9	0.643
40% 1RM L-V	EG	0.793	9	0.581
	CG	0.818	9	0.634
60% 1RM L-V	EG	0.856	9	0.554
	CG	0.892	9	0.498
80% 1RM L-V	EG	0.871	9	0.501
	CG	0.926	9	0.385
90% 1RM L-V	EG	0.843	9	0.541
	CG	0.884	9	0.406

**Table A2:** Differences in baseline testing variables using independent samples *t*-tests.

Measurement	*t*-Value	*df*	*p*-Value	SMD_pre_
CMJ height	0.599	11.055	0.561	0.27
SJ height	0.393	12.212	0.701	0.18
EUR	0.604	18	0.553	0.27
RSI	−1.451	18	0.164	−0.65
FFC time	−0.776	18	0.453	−0.34
FFC PF	1.280	18	0.217	0.57
FFC Impulse	−2.219	18	0.047 *	−0.95
FKA	0.260	13.544	0.799	0.12
BRS	−1.812	18	0.087	−0.81
20% 1RM L-V	0.349	18	0.731	0.29
40% 1RM L-V	0.593	18	0.561	0.33
60% 1RM L-V	0.379	18	0.709	0.25
80% 1RM L-V	−0.259	18	0.799	−0.16
90% 1RM L-V	−0.484	18	0.634	−0.37

*: FFC Impulse showed significant difference between the experimental group and the control group at the baseline value (*p* < 0.05).

**Table A3:** Shapiro–Wilk tests of normality of the difference value of pre-post measurement.

Measurement	Group	Statistic	*df*	*p*-Value
CMJ height	EG	0.956	9	0.740
	CG	0.969	9	0.879
SJ height	EG	0.935	9	0.503
	CG	0.860	9	0.076
EUR	EG	0.929	9	0.441
	CG	0.863	9	0.083
RSI	EG	0.913	9	0.299
	CG	0.913	9	0.299
FFC time	EG	0.909	9	0.278
	CG	0.864	9	0.085
FFC PF	EG	0.990	9	0.997
	CG	0.913	9	0.304
FFC Impulse	EG	0.829	9	0.033 *
	CG	0.927	9	0.418
FKA	EG	0.906	9	0.256
	CG	0.924	9	0.387
BRS	EG	0.914	9	0.559
	CG	0.952	9	0.696
20% 1RM L-V	EG	0.961	9	0.658
	CG	0.923	9	0.493
40% 1RM L-V	EG	0.829	9	0.714
	CG	0.905	9	0.663
60% 1RM L-V	EG	0.853	9	0.561
	CG	0.951	9	0.523
80% 1RM L-V	EG	0.841	9	0.745
	CG	0.848	9	0.639
90% 1RM L-V	EG	0.878	9	0.489
	CG	0.936	9	0.745

*: The difference value of pre-post measurement of FFC Impulse does not meet the requirements of the normal distribution test (*p* < 0.05).
